# Photon antibunching in single-molecule vibrational sum-frequency generation

**DOI:** 10.1515/nanoph-2024-0469

**Published:** 2025-01-14

**Authors:** Fatemeh Moradi Kalarde, Francesco Ciccarello, Carlos Sánchez Muñoz, Johannes Feist, Christophe Galland

**Affiliations:** Institute of Physics, Swiss Federal Institute of Technology Lausanne (EPFL), CH-1015 Lausanne, Switzerland; Inria Paris-Saclay and CPHT, Ecole Polytechnique, Institut Polytechnique de Paris, Palaiseau, France; Center of Quantum Science and Engineering, Swiss Federal Institute of Technology Lausanne (EPFL), CH-1015 Lausanne, Switzerland; Condensed Matter Physics Center (IFIMAC), Universidad Autónoma de Madrid, Madrid, Spain; Institute of Fundamental Physics IFF-CSIC, Calle Serrano 113b, 28006 Madrid, Spain; Departamento de Física Teórica de la Materia Condensada, Universidad Autónoma de Madrid, Madrid, Spain

**Keywords:** photonics, nanocavities, single photon source, photon blockade, cavity optomechanics, vibrational spectroscopy

## Abstract

Sum-frequency generation (SFG) enables the coherent upconversion of electromagnetic signals and plays a significant role in mid-infrared vibrational spectroscopy for molecular analysis. Recent research indicates that plasmonic nanocavities, which confine light to extremely small volumes, can facilitate the detection of vibrational SFG signals from individual molecules by leveraging surface-enhanced Raman scattering combined with mid-infrared laser excitation. In this article, we compute the degree of second order coherence (*g* ^(2)^(0)) of the upconverted mid-infrared field under realistic parameters and accounting for the anharmonic potential that characterizes vibrational modes of individual molecules. On the one hand, we delineate the regime in which the device should operate in order to preserve the second-order coherence of the mid-infrared source, as required in quantum applications. On the other hand, we show that an anharmonic molecular potential can lead to antibunching of the upconverted photons under coherent, Poisson-distributed mid-infrared and visible drives. Our results therefore open a path toward bright and tunable source of indistinguishable single photons by leveraging “vibrational blockade” in a resonantly and parametrically driven molecule, without the need for strong light-matter coupling.

## 1 Introduction

Single-photon sources are a key resource for quantum technologies [1] with pivotal applications in quantum computation [2], communications [3], and metrology [4]. The main challenge in this area is to realize a high-rate solid-state photon source producing indistinguishable photons [5], which demands that pure-dephasing makes a negligible contribution to the emission linewidth [6]. Probabilistic approaches to single-photon emission typically leverage an optical nonlinearity in the material (spontaneous parametric down conversion [7] or degenerate four-wave mixing [8]) to generate time-correlated photon pairs from bulk crystals [9] or photonic integrated circuits [10]. The detection of one photon in, e.g., the idler mode heralds a quantum state very close to a single-photon Fock state in the signal mode [1]. This heralding works provided that the probability of photon pair generation per mode is well below unity, a condition that fundamentally limits the brightness of these type of sources [11]. An alternative approach exploits the anharmonic character of quantum emitters to achieve deterministic single-photon emission via photon blockade [12], [13]. Such emitters include trapped atoms or ions [14], and notably solid-state sources such as immobilized molecules [15], quantum dots [16], nanotubes [17], color centers [18], [19], and many other rising low-dimensional materials [11].

In the last decades, molecules have emerged as a particularly promising platform for integrated quantum photonic technologies [21], given their good degree of coherence [22], frequency tunability [23], [24], versatile coupling to photonic structures [25], [26], [27], [28] and strong non-linearity at the single-photon level [29] enabling effects such as the emission of antibunched light [30], [31], four-wave mixing [32] and sum-frequency generation [20], [33].

**Figure 1:**
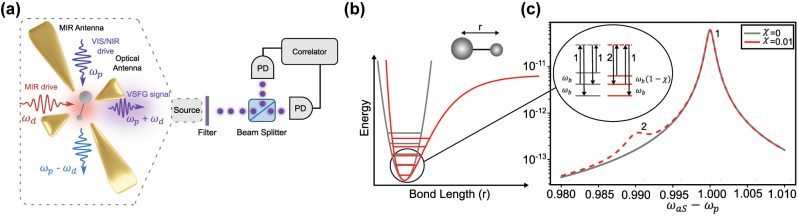
Overview of the proposed scheme. (a) VIS and MIR antennas mediate an efficient interaction between photonic and molecular vibrational modes [20]. A spectrometer or HBT interferometer measures the intensity spectrum or the frequency-filtered second-order correlation of the scattered photons, respectively. (b) Vibrational potential of the molecule considering either a Morse potential (red line) or its harmonic approximation (grey line). (c) As a result of anharmonicity, a second peak (labelled 2) emerges in the thermally activated spontaneous anti-Stokes Raman spectrum, corresponding to the Raman transition from the second to the first excited vibrational levels (see inset). The energy difference between transitions 1 and 2 is noted *χω*_b_, whereby *χ* quantifies the strength of anharmonicity in units of the Raman shift *ω*_*b*_. The thermal occupancy is set here to *n*_th_ = 5 × 10^−3^.

Here, we propose the implementation of a broadly tunable source of single photons based on photon blockade in vibrational sum-frequency generation (VSFG) from a single molecule embedded in a dual-resonant plasmonic nanocavity in the weak-coupling regime [20], [33]. The setup consists in measuring surface-enhanced Raman scattering (SERS) at the single molecule level under simultaneous mid-infrared (MIR) laser excitation, Figure 1a. In contrast to a recent proposal for unconventional photon blockade in a hybrid plasmonic-photonic molecular optomechanical cavity [34], our scheme is operational for arbitrarily weak molecule-cavity coupling strengths and irrespective of the cavity modes quality factors. VSFG is a coherent upconversion process that occurs whenever a vibrational mode that is both Raman- and infrared-active (which requires a non-centrosymmetric molecule) is driven simultaneously with a MIR laser tuned on resonance with the vibrational frequency and a visible or near-infrared (VIS/NIR) laser, typically tuned away from any electronic resonance to avoid parasitic incoherent fluorescence [35], [36], [37]. Three-wave mixing results in upconverted photons at frequencies corresponding to the sum and difference of the incoming laser frequencies; while the difference frequency signal is polluted by spontaneous Raman scattering, VSFG is almost free of incoherent background due to the very low thermal occupancy of the vibration at MIR frequencies, even at room temperature. We will show the feasibility of single-photon generation using VSFG from a single molecule with a sufficient anharmonicity in one of its vibrational modes, resulting in antibunching in the sum-frequency upconverted photons.

Our proposal shares with resonance fluorescence some similitudes regarding the coexistence of coherence (sub-natural linewidth) and single-photon emission, recently explored in several works [38], [39], [40], [41]. Antibunching is revealed at specific frequency windows through the zero-delay second-order correlation function of the frequency-filtered emission [42], [43], [44], which, when analyzed over the parameter space, unveils a complex landscape of interference phenomena between the coherent VSFG nonlinear process and the incoherent anti-Stokes Raman background. This interference is crucial for enabling single-photon emission, which is maximized when the MIR laser is resonant with the vibrational mode and the coherent and incoherent peaks overlap. These results establish our proposed molecular scheme as a novel, privileged platform for studying the role of interference in single-photon emission, closely tied to the notions of conventional and unconventional photon blockade [39].

The versatility of our scheme, together with the picosecond relaxation times of vibrational excitations, promises a valuable tool for the production of indistinguishable single photons at THz repetition rates, enabling a breadth of applications such as the realisation of multi-photon entanglement in boson sampling [45] and cluster states [46], [47], [48], [49]. Furthermore, our observation of the rich phenomenology imprinted in the photon correlations by the nonlinear molecular dynamics suggests promising prospects for quantum Raman spectroscopy boosting analytical, material, and biomedical applications [50], [51], [52], [53]; quantum estimation of molecular parameters and quantum states [54]; and quantum sensing [55].

In the following, we provide a numerical analysis of VSFG based on the scenario presented in [20], [33] in the single or few-molecule limit, focusing on a more rigorous treatment of classical and quantum coherence in the upconversion process and introducing the anharmonicity of the vibrational mode [56]. For the interaction of the molecular vibration with visible light, we adopt an optomechanical description of Raman scattering [57], [58], [59]. We first confirm that, in the harmonic approximation for the vibrational potential, the first- and second-order coherence of the infrared signal are preserved in the upconverted signal, as required for quantum frequency conversion [60], [61] and MIR single-photon spectroscopy [62], [63], [64], [65], [66], [67], [68], [69], [70], [71], [72]. Second, we demonstrate the feasibility of generating antibunched photons under coherent MIR and VIS excitation of a single molecule having a strong enough anharmonicity in its vibrational potential [73], while the molecule remains in the weak coupling regime with both MIR and VIS cavity modes. We conclude by exploring the effect of the number of involved molecules on the degree of antibunching.

## 2 Results

### 2.1 Model

We consider a single vibrational mode of a single molecule placed in a dual-resonant plasmonic cavity featuring two resonances: one in the MIR at *ω*_IR_ ≃ *ω*_b_, where *ω*_b_ is the transition frequency between the ground and first vibrational excited state, and one in the VIS/NIR domain at frequency *ω*_c_ that is considered much higher than the vibrational frequency and sufficiently lower than the first electronic transition of the molecule. The nanocavity is driven by two continuous monochromatic laser beams: one at a MIR frequency *ω*_d_ near-resonant with the cavity mode and molecular vibration, *ω*_d_ ≃ *ω*_IR_ ≃ *ω*_b_, and one at a VIS/NIR frequency *ω*_p_ ≃ *ω*_c_, generating a VSFG signal at a frequency *ω*_p_ + *ω*_d_. The setup is depicted in Figure 1a.

The Hamiltonian of the molecule, restricted to the ground electronic state and the few first vibrational states, is expressed as:

(1) $$H_{\text{mol}}=\omega_{\text{b}}b^{†}b-\frac{\chi }{2}\omega_{\text{b}}b^{†}b^{†}bb.$$

Here and in the following, we choose units where the reduced Planck constant *ℏ* = 1 and we set *ω*_b_ as the energy unit. The vibrational mode (phonon) of the molecule at frequency *ω*_b_ has a corresponding annihilation operator denoted as *b*. The parameter *χ* characterizes the anharmonicity of the vibrational mode. The eigenstates of *H*_mol_ are the Fock states *b* ^†^ *b*|*n*⟩ = *n*|*n*⟩, with energies given by $E_{n}=n\omega_{\text{b}}\left[1-\chi \left(n-1\right)/2\right]$ . We will restrict ourselves to small, realistic values *χ* ≤ 5 × 10^−2^ [73], for which the anharmonicity is only a small correction to the eigenenergies of the first few excited states. A recent theoretical extension of molecular optomechanics discussed more comprehensively how to treat the vibrational anharmonicity [74].

A simple Hamiltonian can be formulated to describe the full system, which takes into account the molecular vibration, the two resonances of the nanocavity, the incident lasers (Ω_IR_ and Ω_c_ denote their respective drive strengths), as well as the resonant dipolar coupling between the MIR cavity mode and the molecular vibration, and its optomechanical (Raman) coupling with the VIS/NIR mode [20].

(2) $$\begin{array}{ll} H & =H_{\text{mol}}+\omega_{\text{IR}}a_{\text{IR}}^{†}a_{\text{IR}}+\omega_{\text{c}}a_{\text{c}}^{†}a_{\text{c}}\\ & \;+i\Omega_{\text{IR}}\left(a_{\text{IR}}^{†}{\text{e}}^{-\text{i}\omega_{\text{d}}t}-a_{\text{IR}}{\text{e}}^{\text{i}\omega_{\text{d}}t}\right)\\ & \;+i\Omega_{\text{c}}\left(a_{\text{c}}^{†}{\text{e}}^{-\text{i}\omega_{\text{p}}t}-a_{\text{c}}{\text{e}}^{\text{i}\omega_{\text{p}}t}\right)\\ & \;+{\tilde{g}}_{\text{IR}}\left(a_{\text{IR}}^{†}+a_{\text{IR}}\right)\left(b^{†}+b\right)+{\tilde{g}}_{\text{c}}a_{\text{c}}^{†}a_{\text{c}}(b^{†}+b). \end{array}$$

We assume decay rates of *κ*_c_, *κ*_IR_, and *γ* for the optical, infrared, and phonon modes, respectively. Following the approximations detailed in Appendix C, we obtain the linearized Hamiltonian

(3) $$\begin{array}{ll} H & =H_{\text{mol}}+\left(\omega_{\text{c}}-\omega_{\text{p}}\right)\delta a_{\text{c}}^{†}\delta a_{\text{c}}\\ & \;+{\tilde{g}}_{\text{c}}\left(\alpha_{\text{c}}\delta a_{\text{c}}^{†}+\alpha_{\text{c}}^{⋆}\delta a_{\text{c}}\right)\left(b^{†}+b\right)\\ & \;+{\tilde{g}}_{\text{IR}}\left(\alpha_{\text{IR}}^{⋆}b{\text{e}}^{\text{i}\omega_{\text{d}}t}+\alpha_{\text{IR}}b^{†}{\text{e}}^{-\text{i}\omega_{\text{d}}t}\right), \end{array}$$

where $\alpha_{\text{IR}}=\frac{\Omega_{\text{IR}}}{i\Delta_{\text{IR}}+\frac{\kappa_{\text{IR}}}{2}}$ and $\alpha_{\text{c}}=\frac{\Omega_{\text{c}}}{i\Delta_{\text{c}}+\frac{\kappa_{\text{c}}}{2}}$ are the intracavity field amplitudes, with Δ_IR_ = *ω*_IR_ − *ω*_d_ and Δ_c_ = *ω*_c_ − *ω*_p_ the cavity-laser detunings. The annihilation operator *δa*_c_ denotes fluctuations in the VIS/NIR mode, while the MIR mode operator is eliminated and only the coherent amplitude *α*_IR_ is retained. The decay rates *κ*_c_ and *γ* apply to *δa*_c_ and *b*, respectively.

In case of zero detuning of the lasers from the cavity resonances (such that *α*_IR_ and *α*_c_ are real), the Hamiltonian simplifies to

(4) $$\begin{array}{ll} H & =\omega_{\text{b}}b^{†}b-\frac{\chi }{2}\omega_{\text{b}}b^{†}b^{†}bb+g_{\text{c}}\left(\delta a_{\text{c}}^{†}+\delta a_{\text{c}}\right)\left(b^{†}+b\right)\\ & \;+g_{\text{IR}}\left(b{\text{e}}^{\text{i}\omega_{\text{d}}t}+b^{†}{\text{e}}^{-\text{i}\omega_{\text{d}}t}\right), \end{array}$$

where we have defined the effective (laser-driven) coupling rates $g_{\text{IR}}={\tilde{g}}_{\text{IR}}\alpha_{\text{IR}}$ and $g_{c}={\tilde{g}}_{c}\alpha_{c}$ .

The Lindblad master equation corresponding to the Hamiltonian in Eq. (4) with the Lindblad dissipation terms due to the decay rates mentioned above is solved using QuTiP [75], [76]. The parameters are fixed in dimensionless units by normalizing all energies to the vibrational frequency *ω*_b_, which typically takes values of tens of THz in localized molecular vibrations. The values set throughout the article are presented in Table 1, unless stated otherwise. The values of the damping rates for plasmonic and vibrational excitations match a recent experimental realization of nanocavity-enhanced SFG [33] (see Table 2), with the main difference that we choose a lower quality factor of the VIS/NIR plasmonic mode. The effective coupling rates *g*_IR_ and *g*_c_ are kept significantly smaller than the inferred single-molecule experimental values from Ref. [33] (see Table 2) because of computational limitations in the size of the total Hilbert space. We discuss further in Appendix D what are the experimentally achievable values of *g*_IR_ and *g*_c_ and the expected upconverted photon rates.

**Table 1:** Default parameters used in the article, unless stated otherwise.

*ω*_b_	*ω*_d_	*γ*	*κ*_c_	*κ*_IR_	Δ_c_	*n*_th_	*g*_IR_	*g*_c_
1	1	10^–3^	4	0.3	0	10^–4^	10^–5^	10^–5^

The dimensionless thermal occupancy is set as $n_{\text{th}}={\left(e^{\frac{\omega_{\text{b}}}{k_{\text{B}}T}}-1\right)}^{-1}$ , where *k*_B_ is the Boltzmann constant and *T* is the temperature. Numerical resolution of the Lindblad equation requires to truncate the Hilbert sub-spaces of each bosonic mode; convergence was achieved for a large enough range of driving strengths with the following dimensions: *q*_c_ = 4 for the VIS/NIR cavity, *q*_b_ = 5 for vibrational mode. Since the Hamiltonian is time-dependent but periodic, we obtain a Floquet expansion of the steady state, $\rho_{s}\left(t\right)=\sum_{n=-n_{b}}^{n_{b}}\rho_{n}{\text{e}}^{\text{i}n\omega_{d}t}$ , truncated at *n*_b_ = 4 terms. When computing the spectrally resolved VSFG intensity, the linewidth of the two-level system acting as a filter is set to Γ_f_ = 10^−5^ and its coupling strength to *ϵ* = 10^−6^. These parameters are modified to Γ_f_ = 10^−2^ and *ϵ* = 10^−5^ when computing spectrally-resolved *g* ^(2)^(0).

### 2.2 Harmonic vibration

**Figure 2:**
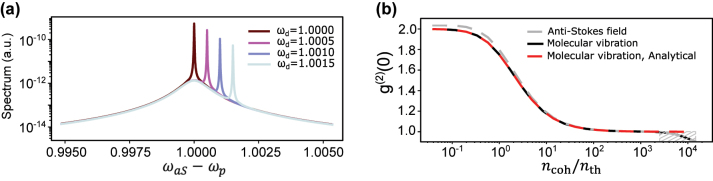
VSFG under harmonic vibrational potential. (a) Anti-Stokes spectra at various mid-infrared laser frequencies. The sharp peak, with its frequency shifting in accordance with the MIR laser frequency, exhibits a linewidth that corresponds to the filter’s characteristics. (b) Second-order correlation function of anti-Stokes photons, $g_{\omega_{\text{aS}},\Gamma_{\text{f}}}^{\left(2\right)}\left(0\right)$ , and molecular vibrations, $g_{b}^{\left(2\right)}\left(0\right)$ , computed both numerically and analytically, plotted versus the ratio of coherent population over thermal population. The coherent population is defined in Eq. (11) and relates to MIR drive strength quadratically. No anharmonicity is assumed (*χ* = 0). Dashed area shows the interval where the truncated Hilbert space fails to correctly approximate the infinite number of levels of a harmonic oscillator.

We begin with results obtained under the harmonic potential approximation for the vibrational mode, i.e., *χ* = 0. Vibrational Raman (anti-Stokes side of the VIS/NIR laser) and VSFG spectra are shown in Figure 2a for various MIR laser frequencies. Each spectrum features a broad and MIR-laser-independent peak corresponding to thermally-activated spontaneous Raman scattering. For increasing VIS/NIR laser power, this peak can grow in intensity through vibrational pumping [77], [78], which is a consequence of quantum back-action in the formalism of molecular cavity optomechanics used here [79]. In our study, we keep the VIS/NIR laser power low enough that this effect is negligible.

On top of the broad anti-Stokes Raman peak, the coherently upconverted VSFG signal at a frequency *ω*_p_ + *ω*_d_ can be seen. Since the driving lasers in our simulation have perfectly defined frequencies, the linewidth of this signal is set only by the linewidth of the filter (see Appendix B), while its frequency is set by energy conservation to the sum of the MIR and VIS/NIR laser frequencies. This behavior was experimentally evidenced in Ref. [33]. In practice, the lineshape of the VSFG signal is expected to be a convolution of those of the two driving lasers.

In order to quantify the photon or phonon statistics and second-order coherence properties of the fields we compute the second-order correlation function at zero time delay, defined for a particular mode *j* as:

(5) $$g_{j}^{\left(2\right)}\left(\tau =0\right)=\frac{〈j^{†}j^{†}jj〉}{{〈j^{†}j〉}^{2}}.$$

Throughout this paper, whenever we refer to the *g* ^(2)^(0) of molecular vibrations (phonons), we apply the formula given above. Eq. (5) disregards the frequency content of the mode and the resulting correlation function is frequency-blind. It is problematic when studying the statistics of the cavity field fluctuations since they contain both Stokes and anti-Stokes scattering contributions, only the latter being of interest here. In order to obtain frequency-resolved photon statistics of the emission of the system, we employ the method proposed in Ref. [42], consisting of including explicit detectors with Lorentzian frequency responses in the simulation.

More explicitly, to compute the cross-correlation between the frequency components *ω*_1_ and *ω*_2_ of the cavity mode fluctuations *δa*_c_, the following Hamiltonians are added to the system’s Hamiltonian in Eq. (4):

(6) $$\begin{array}{c} H_{\text{Sens}}=\omega_{1}\zeta_{1}^{†}\zeta_{1}+\omega_{2}\zeta_{2}^{†}\zeta_{2},\\ H_{\text{Coupl}}=\epsilon \left(\delta a_{c}\zeta_{1}^{†}+\delta a_{c}^{†}\zeta_{1}\right)+\epsilon \left(\delta a_{c}\zeta_{2}^{†}+\delta a_{c}^{†}\zeta_{2}\right), \end{array}$$

where *ζ*_1_ and *ζ*_2_ are bosonic annihilation operators of the two level systems, having decay rates of Γ_f1_ and Γ_f2_ respectively which define the filters linewidth in our model. To maintain the system’s solution unperturbed, *ϵ* must be sufficiently small, satisfying the condition $\epsilon \ll \sqrt{\frac{\gamma \cdot \min\left\{\Gamma_{\text{f}1},\Gamma_{\text{f}2}\right\}}{2}}$ . Subsequently, $g_{\delta a_{c},\Gamma_{f1},\Gamma_{f2}}^{\left(2\right)}\left(\omega_{1},\omega_{2},\tau =0\right)$ is calculated as follows:

(7) $$g_{\delta a_{c},\Gamma_{f1},\Gamma_{f2}}^{\left(2\right)}\left(\omega_{1},\omega_{2},\tau =0\right)=\frac{\left\langle \zeta_{1}^{†}\zeta_{1}\zeta_{2}^{†}\zeta_{2}\right\rangle }{\left\langle \zeta_{1}^{†}\zeta_{1}\right\rangle \left\langle \zeta_{2}^{†}\zeta_{2}\right\rangle }.$$

For simplicity, we will later omit the subscript *δa*_c_, as it is the only mode to which the frequency-resolved method is applied. Additionally, since we only compute the auto-correlation at the anti-Stokes frequency *ω*_aS_ we introduce the following notation:

(8) $$g_{\omega_{\text{aS}},\Gamma_{\text{f}}}^{\left(2\right)}\left(0\right)\equiv g_{\delta a_{c},\Gamma_{f},\Gamma_{f}}^{\left(2\right)}\left(\omega_{\text{aS}},\omega_{\text{aS}},\tau =0\right).$$

Figure 2b shows that as the MIR drive strength increases, both the vibrational mode and the VSFG field exhibit a transition from a thermal state to a coherent state. In this figure, the *x*-axis represents a dimensionless quantity, defined later by Eq. (10) and Eq. (11), which scales linearly with the MIR laser power. The *x*-axis values are derived by sweeping over the MIR drive strength while keeping other parameters fixed. At low enough MIR drive, the vibrational mode is in thermal equilibrium and the anti-Stokes signal is dominated by spontaneous Raman scattering that inherits the same thermal statistics corresponding to *g* ^(2)^(0) = 2 [80]. As the MIR drive increases, the coherent contribution of VSFG to the displaced thermal state increases and *g* ^(2)^(0) asymptotically reaches 1.

One can gain more insight into this behavior by considering the Hamiltonian of the vibrational mode in the rotating frame.

(9) $$H_{\text{b}}=\left(\omega_{\text{b}}-\omega_{\text{d}}\right)b^{†}b+g_{\text{IR}}\left(b+b^{†}\right).$$

The second term corresponds to a coherent displacement of the initial thermal state. In the special case of zero temperature (*n*_th_ = 0), the solution for the vibrational mode is a coherent state |*β*⟩ of amplitude.

(10) $$\beta =\frac{g_{\text{IR}}}{-\left(\omega_{\text{b}}-\omega_{\text{d}}\right)+i\frac{\gamma }{2}}.$$

It is convenient to define a coherent population *n*_coh_ as

(11) $$n_{\text{coh}}\equiv |\beta {|}^{2},$$

which corresponds to the average number of excitations in the vibrational mode when *g*_c_ = 0 and *n*_th_ = 0.

In the general case of arbitrary *n*_th_, the mean vibrational population is given by

(12) $$n_{\text{b}}=\mathrm{Tr}\left\{\rho_{\text{th}}\;\left(b^{†}+\beta^{⋆}\right)\left(b+\beta \right)\right\}=n_{\text{th}}+{\left|\beta \right|}^{2},$$

with

$$\rho_{\text{th}}=\overset{\infty }{\underset{n=0}{\sum }}P_{n}|n〉〈n|\;\text{with}\;P_{n}=\frac{n_{\text{th}}^{n}}{{\left(1+n_{\text{th}}\right)}^{n+1}}.$$

The non-normalized second-order correlation is given by:

$$\mathrm{Tr}\left\{\rho_{\text{th}}\;{\left(b^{†}+\beta^{⋆}\right)}^{2}{\left(b+\beta \right)}^{2}\right\}=2n_{\text{th}}^{2}+4n_{\text{th}}{\left|\beta \right|}^{2}+{\left|\beta \right|}^{4}$$

and after normalization

(13) $$g_{\text{b}}^{\left(2\right)}\left(0\right)=2-\frac{{\left|\beta \right|}^{4}}{{\left(n_{\text{th}}+{\left|\beta \right|}^{2}\right)}^{2}}=2-\frac{1}{{\left(1+\frac{n_{\text{th}}}{n_{\text{coh}}}\right)}^{2}},$$

which is the analytical expression plotted as a dashed red curve in Figure 2b. The hashed area at high *n*_coh_ shows the domain where the Hilbert space truncation causes inaccuracies in the numerical solution. We therefore limit future calculations to MIR powers below this value.

### 2.3 Anharmonicity in the vibration

In reality, vibrational modes of small molecules have non-negligible anharmonicities [81], responsible for temperature-dependent Raman shifts and lineshapes [82] and for the observation of so called ‘hot bands’ in Raman scattering when the anharmonicity is larger than the peak linewidth [73].

**Figure 3:**
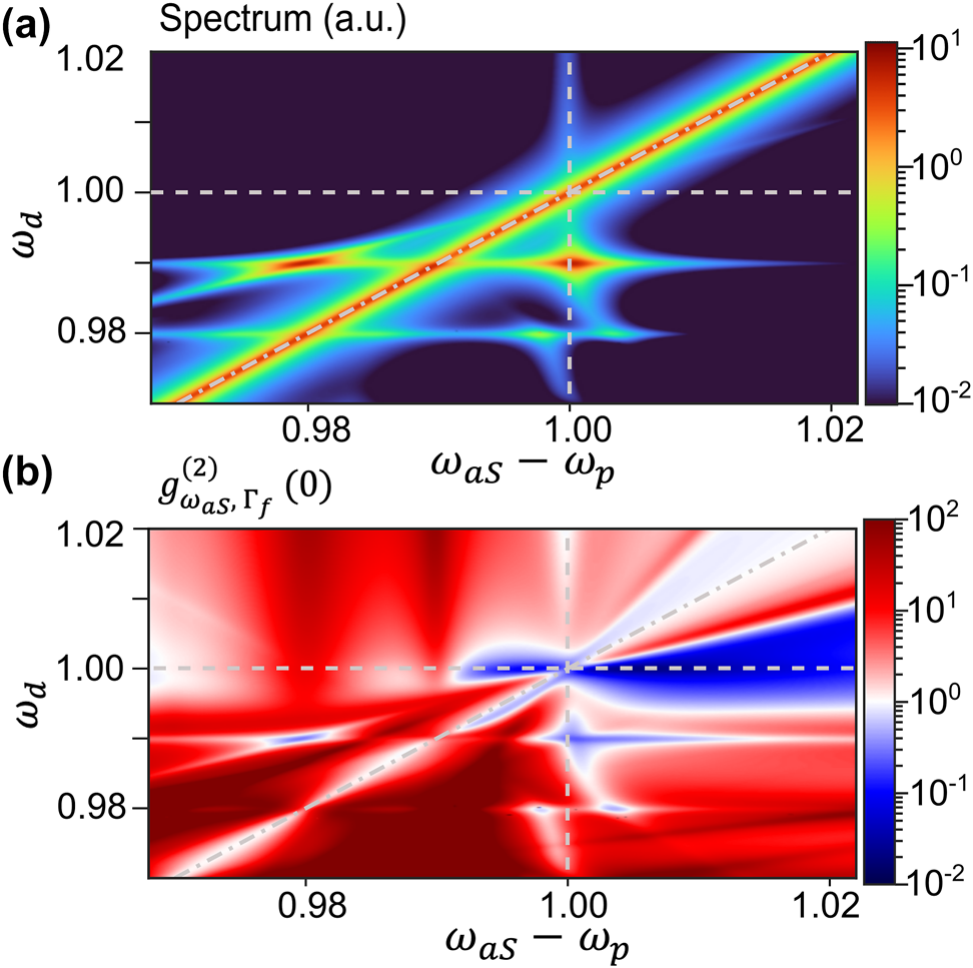
VSFG under anharmonic vibrational potential. Maps of the (a) emission intensity and (b) second-order correlation function of the filtered anti-Stokes field as a function of the MIR driving frequency *ω*_d_ and VIS/NIR emission Raman shift *ω*_aS_ − *ω*_p_, under strong MIR driving (*g*_IR_ = 10^−4^ or equivalently *n*_coh_ = 0.04 for *ω*_d_ = 1). The dot-dashed grey lines indicate *ω*_d_ = *ω*_aS_ − *ω*_p_.

The main insight of this work is that the presence of this anharmonicity introduces rich features in the spectral properties of the emission, particularly in its statistics. This is shown in Figure 3, where we plot both the spectrum and the frequency-resolved second-order correlation, $g_{\omega_{\text{aS}},\Gamma_{\text{f}}}^{\left(2\right)}\left(0\right)$ , as a function of the driving frequency *ω*_d_. Here, the anharmonicity parameter is set to *χ* = 0.02, which is typical of vibrational modes in small organic molecules used in SERS (see Appendix E), and the filter linewidth is kept equal to Γ_f_ = 10^−3^ in both panels for direct comparison. The dot-dashed grey lines indicate the frequency of the VSFG signal, confirming that it remains the most important contribution to the emission. Nevertheless, the anharmonicity enables multiphoton excitation processes that imprint new features on the spectrum. In particular, when two-photon absorption to the second excited state is resonant, at *ω*_d_ = 0.99, additional double-peaked emission appears at the transition frequencies between levels 1 → 0 (*ω*_aS_ − *ω*_p_ = 1) and 2 → 1 (*ω*_aS_ − *ω*_p_ = 0.98).

Regarding photon statistics, the VSFG signal is observed to be antibunched provided that it overlaps with the incoherent anti-Stokes background, i.e., that the driving frequency is close to the vibrational transition, *ω*_d_ ≈ *ω*_b_. This establishes that the incoherent background due to the anti-Stokes process must be present to obtain strong antibunching, in accordance with the recent understanding of antibunching in resonance fluorescence as a consequence of interference between coherent and incoherent terms [38], [39], [40], [41]. A second prominent antibunching feature is observed under driving at the two-photon resonance *ω*_d_ = 0.99, which leads to antibunched emission at the two frequencies *ω*_aS_ − *ω*_p_ = {0.98, 1}. Moreover, the frequency-resolved cross-correlation measured at these two frequencies (not shown) reveals a large bunching, *g* ^(2)^(*ω*_aS,1_ − *ω*_p_ = 1, *ω*_aS,2_ − *ω*_p_ = 0.98) ≈ 8, indicating that these photons are produced in pairs through cascaded emission. This could be exploited for the generation of entangled photon pairs in the VIS or NIR range, which could be potentially useful for quantum communication protocols.

Further understanding of this phenomena and its dependence on the anharmonicity *χ* can be obtained by analyzing the fluctuations of the vibrational degree of freedom. Under the assumption of *g*_IR_ ≪ *γ*, *g*_c_ = 0 and *n*_th_ = 0, the second-order coherence of the resonantly driven vibrational mode has an analytical expression (see, for example, Ref. [39]):

(14) $$g_{\text{b}}^{\left(2\right)}\left(0\right)=\frac{\gamma^{2}+4{\left(\omega_{\text{b}}-\omega_{\text{d}}\right)}^{2}}{\gamma^{2}+{\left[2\left(\omega_{\text{b}}-\omega_{\text{d}}\right)-\chi \omega_{\text{b}}\right]}^{2}}.$$

**Figure 4:**
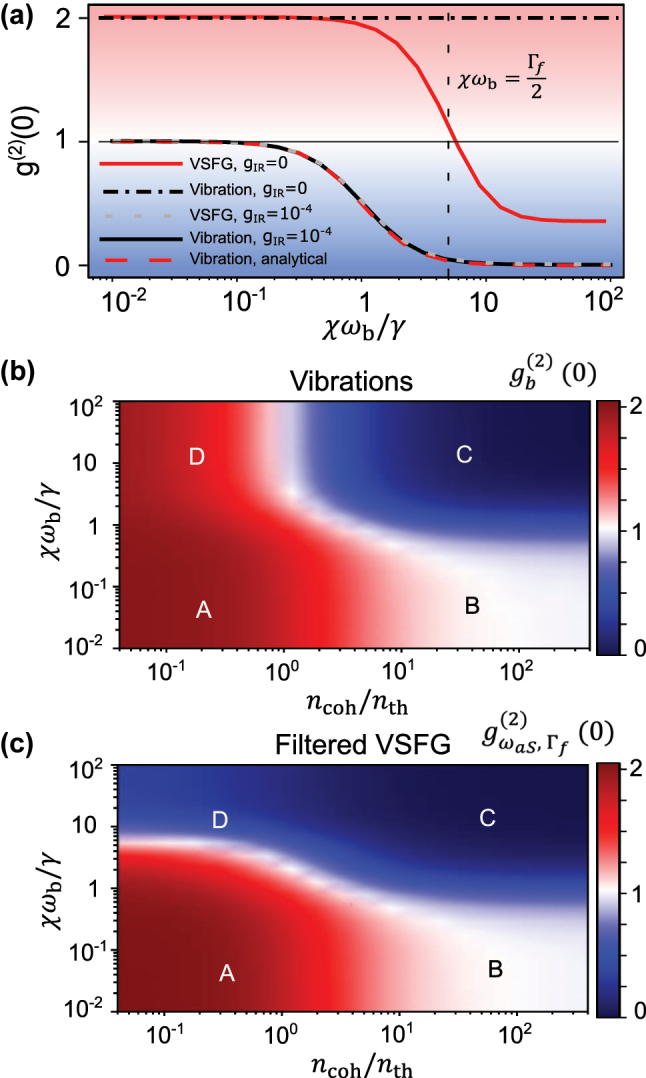
Regimes of statistics of VSFG (a) evolution of frequency-blind second-order correlation function of the vibrational mode, $g_{b}^{\left(2\right)}\left(0\right)$ , and of the frequency-filtered second-order correlation function of the anti-Stokes field, $g_{\omega_{\text{aS}},\Gamma_{\text{f}}}^{\left(2\right)}\left(0\right)$ , plotted against the relative strength of anharmonicity, both in the absence (*g*_IR_ = 0) and in the presence (*g*_IR_ = 10^−4^ or equivalently *n*_coh_ = 0.04) of MIR drive. The analytical curve is from Eq. (14). (b,c) Second-order correlation function of vibrations, $g_{b}^{\left(2\right)}\left(0\right)$ , in (b) and filtered anti-Stokes field, $g_{\omega_{\text{aS}},\Gamma_{\text{f}}}^{\left(2\right)}\left(0\right)$ , in (c) plotted versus the dimensionless strengths of MIR drive and anharmonicity. Capital letters identify four regions of parameters discussed in the text.

This expression is shown as a dashed red curve in Figure 4a, against the dimensionless parameter *χω*_b_/*γ*, showing excellent agreement with the numerical computation of $g_{\text{b}}^{\left(2\right)}\left(0\right)$ using the full Lindblad equation (solid black line). The figure also shows that the second-order correlation of the anti-Stokes field filtered around the VSFG frequency under high MIR power (dashed grey line) very closely follows $g_{\text{b}}^{\left(2\right)}\left(0\right)$ . This is expected, given the ‘beam-splitter’ form of the optomechanical Hamiltonian for the anti-Stokes sideband (see Appendix A).

Most importantly, all calculations confirm that when the anharmonicity is larger than the vibrational linewidth, *χω*_b_ > *γ*, the filtered anti-Stokes signal becomes strongly antibunched. This condition can also be written *χ* > 1/*Q*_m_ where *Q*_m_ is the quality factor of the vibration. However, it should be noted that, even though the main emission line is coherent and narrow (limited by the filter linewidth), antibunching is only observed if the emission from a sufficiently broad frequency interval is included (see Figure 7 in Appendix B), i.e., if the filter is not too narrow and the incoherent background is included. Therefore, joint narrow linewidths and single-photon emission cannot be straightforwardly obtained, as has been recently illustrated in resonance fluorescence in driven two-level systems [38], [39], [40], [41].

This result should be compared with the curves obtained without MIR drive plotted with solid red and dot-dashed black lines in Figure 4a, corresponding to the case of spontaneous Raman scattering from a single molecule. In this case, while the vibrational mode remains in thermal equilibrium, it is also possible to observe photon antibunching in the spectrally filtered anti-Stokes field, but only under the intuitive condition that the anharmonicity exceeds the filter linewidth Γ_f_. There are two other major drawbacks with this undriven approach: First, the spontaneous anti-Stokes photon flux can only be increased by increasing the VIS/NIR pump power, which more readily induces damage to the nanocavity [83]. Second, the first-order coherence property of the anti-Stokes field is dictated by that of the molecular vibration, for which the linewidth *γ* can have non-negligible contributions from pure dephasing, thereby degrading the indistinguishability of the antibunched photons.

In order to capture the different regimes of photon statistics for the upconverted signal, we plot in Figs. 4b and c the second-order correlation function for the vibration and the filtered anti-Stokes field as functions of $\frac{n_{\text{coh}}}{n_{\text{th}}}$ and $\frac{\chi \omega_{\text{b}}}{\gamma }$ . Overall, these color maps show that antibunching of the filtered anti-Stokes field is achieved by having strong enough MIR drive and large enough anharmonicity (region C in both plots). For weak anharmonicity (regions A and B), an increase in MIR drive causes a transition from a thermal to a coherent state for both the vibration and the anti-Stokes field, as already discussed in Figure 2b. Finally, in region D, where the anharmonicity is large but the MIR drive is weak, the vibrational mode remains in thermal equilibrium but the filtered anti-Stokes photons may become antibunched as a result of rejecting all photons originating from Raman transitions beyond the ground to first excited vibrational state. The benefit of the MIR drive in our scheme is evidenced in Figure 4c by the higher degree of antibunching in region C with less stringent requirements on the magnitude of anharmonicity, compared to region D.

### 2.4 Few molecules

Achieving single molecule Raman spectroscopy remains a nontrivial experimental task despite multiple demonstrations since its first observation in 1997 [84]. We therefore investigate whether antibunching persists in the presence of several molecules that contribute to VSFG. In the limit of a large number of molecules, collective vibrational excitations are expected to become perfectly harmonic, which is a general result for an ensemble of two-level systems [85]. But in our system the increase in the number of molecules leads to an increase in the collective resonant (MIR) and optomechanical (VIS/NIR) coupling strengths, potentially resulting in the excitation of higher excited vibrational states thereby recovering the anharmonicity. To clarify the expected trend, we perform complete numerical evaluation of the model with *N* = 1, 2 and 3 molecules. For simplicity, we consider that all molecules are identical and have the same coupling rates to the common nanocavity modes:

(15) $$\begin{array}{ll} H_{\text{N}} & =\;\left(\omega_{\text{c}}-\omega_{\text{p}}\right)\delta a_{\text{c}}^{†}\delta a_{\text{c}}+\overset{N}{\underset{i=1}{\sum }}\omega_{\text{b}}b_{i}^{†}b_{i}-\frac{\chi }{2}\omega_{\text{b}}b_{i}^{†}b_{i}^{†}b_{i}b_{i}\\ & \;+\overset{N}{\underset{i=1}{\sum }}g_{\text{c}}\left(\delta a_{\text{c}}^{†}+\delta a_{\text{c}}\right)\left(b_{i}^{†}+b_{i}\right)\\ & \;+\overset{N}{\underset{i=1}{\sum }}g_{\text{IR}}\left(b_{i}{\text{e}}^{\text{i}\omega_{\text{d}}t}+b_{i}^{†}{\text{e}}^{-\text{i}\omega_{\text{d}}t}\right). \end{array}$$

**Figure 5:**
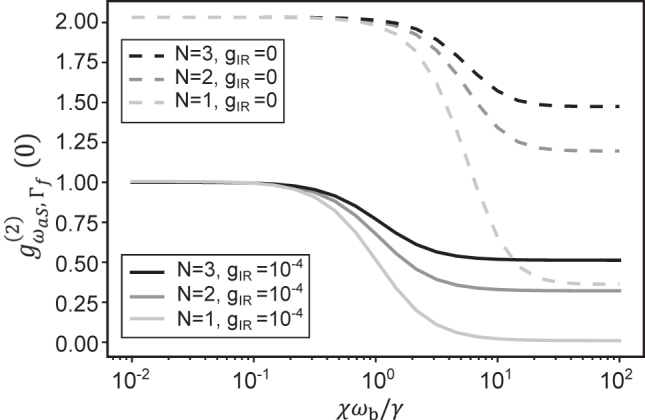
Frequency-filtered second-order correlation function of the anti-Stokes field, $g_{\omega_{\text{aS}},\Gamma_{\text{f}}}^{\left(2\right)}\left(0\right)$ , plotted versus anharmonicity for different number of molecules.

**Figure 7:**
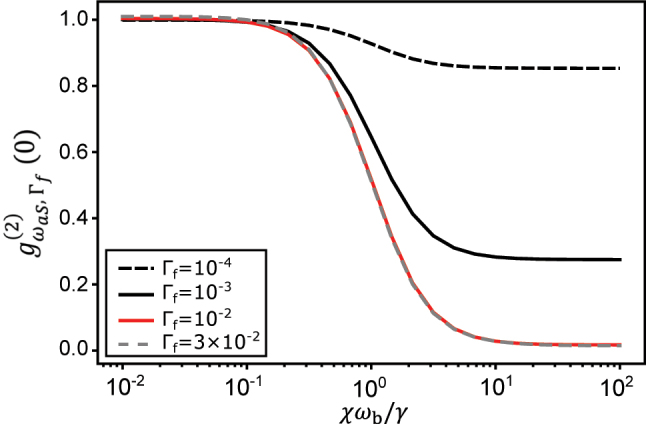
Variation of the second-order correlation function of the filtered anti-Stokes field $g_{\omega_{\text{aS}},\Gamma_{\text{f}}}^{\left(2\right)}\left(0\right)$ versus the strength of anharmonicity in the high MIR drive strength regime (*g*_IR_ = 10^−4^ or *n*_coh_ = 0.04), considering different filter linewidths Γ_f_. We recall that *γ* = 10^−3^.

The second-order correlation *g* ^(2)^(0) for the filtered anti-Stokes field obtained from the solution to the corresponding Lindblad master equation is plotted in Figure 5 for *N* = 1, 2, 3, with (solid lines) and without (dashed lines) an MIR drive. The results confirm the intuition from the large *N* limit: the degree of achievable antibunching quickly decreases as the number of molecules participating in Raman scattering and VSFG increases. Interestingly, we find that this decrease cannot be compensated by arbitrarily increasing the anharmonicity of each molecule as characterized by the parameter *χ*. This can be attributed to the fact that for large enough anharmonicity, each vibration acts as a two-level system, and the collective behavior of the ensemble is dominated by the harmonic limit mentioned above.

## 3 Conclusions

We proposed a new scheme to produce antibunched photons by performing vibrational sum-frequency generation on a single molecule. Our method leverages the anharmonicity naturally present in the potential of vibrational modes that are localized on few molecular bonds to realize a form of conventional photon blockade without the need for strong light-matter coupling. Our calculations show that under sufficiently strong MIR drive, characterized by the ratio of coherent to thermal vibrational population $\frac{n_{\text{coh}}}{n_{\text{th}}}\geq 10$ , almost complete antibunching is achieved whenever the anharmonicity satisfies *χω*_b_ ≥ *γ*, i.e., the energy difference between the first and second vibrational transitions is larger than the level linewidth. This condition is expected to be realized for small molecules typically used in SERS experiments [86] (see Appendix E).

Our proposal is feasible with current experimental capabilities. The main requirement is to be able to measure Raman scattering from a single molecule, which can be achieved in various plasmonic nanoantennas and scanning tip systems [87]. Efficient coupling of MIR light into the system can be achieved using dual-resonant plasmonic antennas [33], [88] or the broadband nanofocusing capabilities of metallic tips [89], [90]. The main foreseen difficulty will be to spectrally and temporally isolate the antibunching dip in the anti-Stokes photon flux: First, the broadband non-resonant SFG coming from the metallic surface and the molecule’s electronic response [91] must be kept smaller than the vibrational contribution to SFG. Second, the experiment should be conducted under picosecond pulsed excitation to resolve the antibunching in time by outcompeting the relaxation of molecular vibrations on a substrate [92].

Under pulsed excitation, it is also possible to make the photon generation scheme deterministic by adjusting the VIS/NIR pulse intensity to reach unity vibration-to-photon conversion efficiency [93]; but it requires that the optomechanical nanocavity is deep in the resolved-sideband regime so as to suppress spontaneous Stokes scattering. Prospective single-photon rates achievable in this regime with our proposed scheme are discussed in Appendix D. The richness and versatility of the proposed scheme may also open perspectives for the generation of entangled photons and quantum metrology applications based on the detection of photon correlations, while offering a spectroscopic method for the characterisation of vibrational anharmonicity at the single-molecule level.

## Figures and Tables

**Figure 6:**
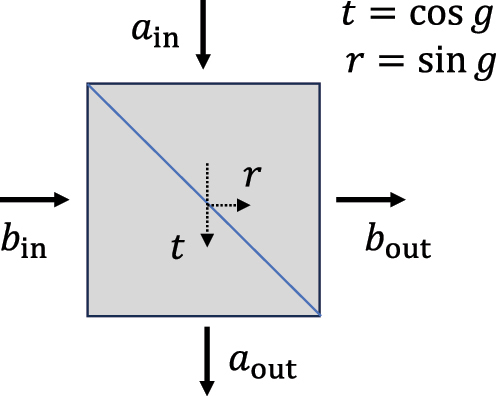
Beam splitter with transmission and reflection coefficients of *t* and *r* and two input arms and two output arms.

**Figure 8:**
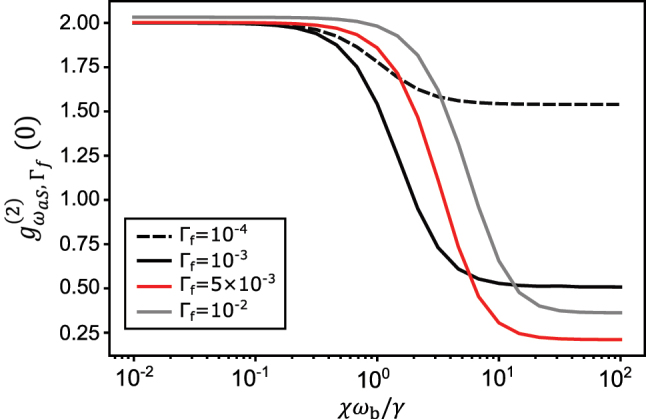
Variation of the second-order correlation function of the filtered anti-Stokes field $g_{\omega_{\text{aS}},\Gamma_{\text{f}}}^{\left(2\right)}\left(0\right)$ versus the strength of anharmonicity in the absence of MIR drive (*g*_IR_ = 0), considering different filter linewidths Γ_f_. We recall that *γ* = 10^−3^.

**Figure 9:**
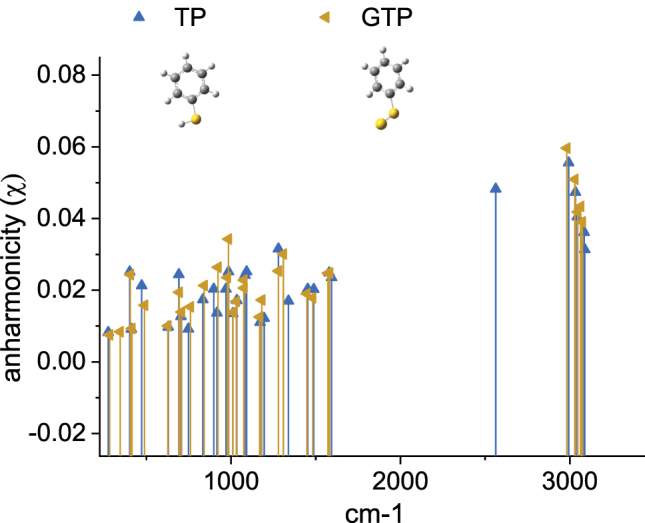
Value of the anharmonicity parameter *χ* computed by DFT for thiophenol (TP, blue triangle) and gold-bound thiophenol (GTP, orange triangle) plotted against the normal mode frequency.

## Appendix A: Beam splitter interaction

Assuming that the optomechanical coupling is small compared to the infrared coupling in Eq. (4), *g*_IR_ ≫ *g*_c_, one can neglect quantum backaction of the cavity mode on the vibration, whose state is then mostly governed by the coupling to the MIR field and to the environment. To estimate the state of optical mode at the anti-Stokes sum-frequency sideband of the VIS/NIR laser, we need to treat the optomechanical coupling term, $g_{\text{c}}\left(\delta a_{\text{c}}^{†}+\delta a_{\text{c}}\right)\left(b^{†}+b\right)$ , which reduces to $g_{\text{c}}\left(\delta a_{\text{c}}^{†}b+\delta a_{\text{c}}b^{†}\right)$ in the rotating wave approximation. It is a bilinear interaction between the two modes and, after changing the notation *δa*_c_ → *a*, the associated unitary operator takes the form

$$U_{\text{BS}}=e^{g\left({\text{e}}^{\text{i}\theta }a^{†}b-{\text{e}}^{-\text{i}\theta }ab^{†}\right)},$$

where *g* = ∥*g*_c_∥ and *θ* = arg  (*g*_c_). It is a general beam splitter interaction as depicted in Figure 6 with the relation between input and output operators given by

(A1)
$$\begin{array}{c} a_{\text{out}}=U_{\text{BS}}^{†}\;a_{\text{in}}\;U_{\text{BS}}=\cos\left(g\right)a_{\text{in}}+\sin\left(g\right){\text{e}}^{\text{i}\theta }b_{\text{in}},\\ b_{\text{out}}=U_{\text{BS}}^{†}\;b_{\text{in}}\;U_{\text{BS}}=\cos\left(g\right)b_{\text{in}}-\sin\left(g\right){\text{e}}^{-\text{i}\theta }a_{\text{in}}, \end{array}$$

where *g* characterizes the beam splitter transmission ratio through *t* = cos *g* and *r* = sin *g*, see Figure 6.

In our context, the input modes of the beam splitter are populated by vacuum fluctuations for the anti-Stokes field *a*_in_ and by the thermal and MIR-driven vibrational state for *b*_in_; the output mode *a*_out_ corresponds to the emitted anti-Stokes field on which the *g* ^(2)^(0) measurement is performed, while *b*_out_ reflects the state of the vibration modified by the anti-Stokes part of the optomechanical interaction, but it is a weak perturbation since we neglect quantum backaction. Finally, a known result is that, when one of the input mode is in a vacuum state, the *g* ^(2)^(0)’s of both outgoing fields from a beam splitter are equal to that of the non-vacuum input port, in particular here $g_{a_{\text{out}}}^{\left(2\right)}\left(0\right)=g_{b_{\text{in}}}^{\left(2\right)}\left(0\right)$ (see e.g. Sec. IV.D in Ref. [94]). In other word, the vibrational mode excitation number statistics is mapped onto the anti-Stokes field generated through the optomechanical (Raman) interaction. The (usually dominant) vacuum noise entering in the process has no impact on photon number detection, as long as dark counts can be neglected [95].

## Appendix B: Impact of filter linewidth on the measured $g_{\omega_{\text{aS}},\Gamma_{\text{f}}}^{\left(2\right)}\left(0\right)$

As investigated in Ref. [96], too narrow spectral filtering may alter the intrinsic photon statistics of a single photon source into that of a thermal state. In our study, under strong MIR-drive, the intensity of the anti-Stokes field is dominated by VSFG over spontaneous Raman scattering, there is no need to aggressively filter the signal; the role of filtering is here to suppress the contribution to *g* ^(2)^ from spontaneous Stokes scattering and VDFG (difference frequency) on the other side of the VIS/NIR laser. We show in Figure 7 that, as found in Ref. [96], a decrease in filter linewidth destroys antibunching in the filtered field. That’s why we chose Γ_f_ = 10*γ* = 10^−2^ for all figures in the main text under MIR drive.

In the absence of MIR drive, photon antibunching is only obtained by rejecting anti-Stokes photons coming from the second (and higher) vibrational transition, which is detuned by *χω*_b_ from the first one, imposing an upper limit on filter linewidth Γ_f_ ≤ *χω*_b_. Besides, the constraint *χω*_b_ ≥ *γ* remains, as for any conventional photon blockade scheme, leading to *γ*, Γ_f_ ≤ *χω*_b_. Finally, as reminded above, filtering below the natural linewidth of the anti-Stokes photons *γ* degrades antibunching by ‘thermalizing’ the photon statistics, imposing Γ_f_ ≥ *γ*. Altogether we obtain the stringent constraints *γ* ≤ Γ_f_ ≤ *χω*_b_, which leaves little room for the choice of Γ_f_ since in practice the anharmonicity is not much greater than *γ*. This parameter space is explored in Figure 8. Overall, for a fixed filter linewidth, the transition to an antibunched state occurs when $\chi \approx \frac{\Gamma_{\text{f}}}{2}$ . But as the filter linewidth is decreased below *γ*, *g* ^(2)^(0) approaches 2 even where antibunching is expected, consistent with the findings of Ref. [96].

## Appendix C: Approximations

To derive Eq. (3), several approximations are employed. First, the rotating wave approximation is applied to the MIR coupling term. Second, both the MIR and VIS/NIR modes are linearized since their coupling rates to the vibration are both assumed to be small compared to all other energies in the Hamiltonian. Consequently, *a*_c_ is expressed as *α*_c_ + *δa*_c_, where *α*_c_ is the solution in the absence of the optomechanical coupling term, representing a coherent state, and *δa*_c_ represents the fluctuations induced by the optomechanical coupling term. Additionally, *a*_IR_ is replaced by *α*_IR_, with *α*_IR_ representing the coherent state in the absence of the MIR coupling term. The fluctuations in this case are neglected for simplicity, as the primary interest lies in the VSFG field properties contained in *δa*_c_.

Next, since the simplified Hamiltonian, Eq. (4), is periodically time-dependent, a Floquet solution of the following form has been considered for the Lindbladian master equation:

(C1)
$$\rho \left(t\right)=\overset{n_{b}}{\underset{n=-n_{b}}{\sum }}\rho_{\text{n}}{\text{e}}^{\text{i}n\omega_{\text{d}}t},$$

where in principle the bounds of the sum must go to infinity. Nevertheless, we truncate at *n*_b_ = 4 and it is verified that considering more terms does not change the results.

## Appendix D: Achievable single photon rates

As explained in the main text, computational costs quickly become prohibitive as the size of the Hilbert space increases. Consequently, when using the single-molecule coupling rates inferred from the experiment of Ref. [33] and summarized here in Table 2, very low laser powers of *P*_IR_ = 20 nW and *P*_c_ = 0.2 nW must be assumed in order to yield the effective coupling rates mentioned in Table 1. We note that these values correspond to the powers effectively coupled to the nanocavity, factoring out the in-coupling efficiency [98]. For such values, the outgoing rate of single photons is computed to be on the order of 100 Hz (again, not accounting for out-coupling efficiency). We now discuss the experimental prospects for higher photon rates.

**Table 2:** Experimental parameters inferred from Ref. [33] for the case where the nanocavity would contain a single molecule. All values are expressed in Hz instead of rad/s, so that factors 2*π* are implicit. Note that the value of *γ* is that measured for the ensemble and includes inhomogeneous broadening; it will likely be smaller for a single molecule, for which coherence times beyond 10 ps have been reported [97].

*ω*_b_	*γ*	${\tilde{g}}_{\text{IR}}$	${\tilde{g}}_{\text{c}}$	*n*_th_	*ω*_d_	*ω*_p_
30 THz	0.3 THz	6 GHz	60 GHz	10^–^^4^	30 THz	300 THz
***ω*_IR_**	***ω*_c_**	***κ*_IR_**	***κ*_c_**			
30 THz	300 THz	10 THz	30 THz			

When increasing the in-coupled MIR power to 300 nW, and for a sufficiently large anharmonicity so that we can use a two-level-system approximation, we compute that the Rabi frequency for the transition between the lowest two vibrational levels is comparable to the vibrational decay rate, indicating the regime of saturation. In this regime, the outgoing single-photon rate is mainly determined by the vibration-to-VIS/NIR photon conversion efficiency through the optomechanical interaction. This conversion efficiency has an analytical formula in the resolved-sideband regime [99] in terms of the optomechanical cooperativity $C_{\text{om}}=\frac{4g_{c}^{2}}{\kappa_{c}\gamma }$ [98]. Currently, the largest values of single-molecule optomechanical cooperativities have been obtained in picocavities; in particular, Ref. [100] claimed the observation of the optical spring effect on a single molecule, implying that $C_{\text{om}}$ can be of order unity in a picocavity. The resulting conversion efficiency would therefore be of order unity as well, limited mainly by the input and output coupling efficiencies [98].

Altogether, the above considerations suggest that the single-photon source proposed here could be made to operate quasi-deterministically under pulsed excitation at moderate laser powers – provided that the optomechanical interaction takes place in the well resolved sideband regime, i.e., *κ* < *ω*_b_. If not, spurious noise from Stokes scattering causing vibrational pumping (quantum backaction) is expected to degrade antibunching for too large optomechanical driving strengths. Finally, we refer to a recent experiment where incoherent MIR to VIS conversion from a nanocavity-embedded single molecule was reported with an efficiency of 0.1 [101] for moderate pump powers. This experiment benefited from the VIS electronic resonance to obtain high anti-Stokes fluorescence rate, at the cost of losing the coherence of the process – which is critical in our proposal. Nevertheless, Ref. [101] suggests that high conversion efficiencies are possible also in the coherent upconversion regime with optimized dual-resonant nanocavities or scanning tip geometries [102], [103] and tailored molecules with high Raman cross-sections [104], [105], [106].

## Appendix E: Magnitude of the anharmonicity for relevant molecules

In this Section, we show that typical organic molecules used in SERS and VSFG experiments feature sufficiently large anharmonicities to fall in the regime of photon antibunching uncovered in the main text. We take thiophenol (TP) as a representative example: it is a small organic molecule (see Figure 9) amenable to precise calculations; moreover, TP self-assembled monolayer (SAM) on gold were experimentally characterized by VSFG, revealing several active vibrational modes in the 1,000–1,100 cm^−1^ window [107]. We perform density functional theory (DFT) calculations on TP and Au-bound thiophenol (GTP) molecules using the Gaussian16 [108] quantum chemistry code. We employ the B3LYP functional with empirical dispersion correction (GD3BJ) due to its accurate performance in anharmonicity calculations documented in Ref. [109], combined with the Def2TZVPPD basis set. Geometry optimization was performed with tight convergence criteria (i.e., 10^−5^ Hartree/Bohr and 4 × 10^−5^ Bohr on RMS forces and displacements, respectively, with thresholds for the maximum values being 1.5 times larger), and the ground state minima were confirmed by Hessian evaluations. Harmonic energies were obtained using analytic second derivatives of the energy, and third and fourth order numerical derivatives. Anharmonic simulations were performed using the GVPT2 model implemented in Gaussian16.

To follow the conventional notation in physical chemistry, we write the energy of the vibrational level *n* of an anharmonic oscillator as:

(E1)
$$E_{\text{n}}=\left(n+\frac{1}{2}\right){\tilde{\omega }}_{\text{b}}-{\left(n+\frac{1}{2}\right)}^{2}{\tilde{\omega }}_{\text{b}}\tilde{\chi }+O\left(n^{3}\right),$$

where ${\tilde{\omega }}_{\text{b}}$ is the harmonic energy of the vibrational quanta and $\tilde{\chi }$ is the anharmonic constant. To find the connection between $\tilde{\chi }$ and the value of *χ* as defined in the main text, we express the frequencies of the 0 → 1 and 1 → 2 transitions in the two formalisms:

(E2)
$$\omega_{0\to 1}={\tilde{\omega }}_{b}\left(1-2\tilde{\chi }\right)=\omega_{b},$$

(E3)
$$\omega_{1\to 2}={\tilde{\omega }}_{b}\left(1-4\tilde{\chi }\right)=\omega_{b}\left(1-\chi \right).$$

From this system of equations we can express

(E4)
$$\chi =\frac{2\tilde{\chi }}{1-2\tilde{\chi }}.$$

For most of the modes, the anharmonic parameter *χ* (as defined in the main text) is between 1 and 5 % (see Figure 9), with the C–H stretching modes characterized by higher anharmonicity, as experimentally observed on methyloxiirane, for example [110]. These values are also compatible with recent experimental findings in SERS [86].
